# The Effects of Urban Natural Environments on Preference and Self-Reported Psychological Restoration of the Elderly

**DOI:** 10.3390/ijerph18020509

**Published:** 2021-01-09

**Authors:** Ling Qiu, Qujing Chen, Tian Gao

**Affiliations:** Department of Landscape Architecture, College of Landscape Architecture and Arts, Northwest A&F University, Yangling 712100, Shaanxi, China; qiu.ling@nwsuaf.edu.cn (L.Q.); jing941001@nwafu.edu.cn (Q.C.)

**Keywords:** urban natural environments, perceived restorativeness, perceived sensory dimensions (PSDs), landscape design

## Abstract

The world is facing the challenge of aging populations. Urban natural environments, including green spaces and blue spaces, have been demonstrated to have great benefits to the mental restoration of the elderly. However, the study of the specific characteristics of urban environments that are popular and the most restorative for the elderly is still lacking. Photo elicitation as visual stimuli was utilized to explore the differences in preference and psychological restoration of the elderly through the perception of the eight perceived sensory dimensions (PSDs) in different types of urban environments. The results showed that: (1) The respondents had different perceptions of the eight PSDs in the different urban natural environments. Blue space and partly-closed green space were more preferred by the elderly, and also had more psychological restorative effects on the elderly. (2) There was no significant correlation between the number of highly perceived PSDs and preference, as well as between the number of highly perceived PSDs and psychological restoration. However, there was a significant correlation between preference and psychological restoration. (3) Partly-closed green space with more Serene and Refuge qualities, and blue space with more Serene, Refuge and Prospect properties were optimal characteristics for psychological restoration of the elderly. In addition, open green space with more Prospect, Serene and Social qualities, and closed green space with more Space, Refuge and less Nature properties could also increase psychological restoration of older adults. These findings can provide useful guidelines for restorative environmental design for the elderly in the future.

## 1. Introduction

The world is facing a dual challenge of aging populations and rapid urbanization. According to the World Health Organization [1], the proportion of the world’s population over 60 years old is predicted to nearly double from 12% to 22% between 2015 and 2050, reaching 2 billion by 2050. However, urbanization has resulted in increasing amounts of natural environments being replaced by built-up areas, and people have thus reduced their exposure to nature, which can cause urban inhabitants, especially the elderly, face serious physical and mental health problems [2]. Older adults already have reduced activity and restricted travel due to the deterioration of their physical functions [3,4], and are more vulnerable to health problems than other age groups. Therefore, being close in proximity to urban natural environments is particularly important for their overall health and daily life.

People are traditionally attracted to nature and the relationship between natural environments and human health has been a wide concern for environmental psychologists since 1980s. There are two main theories which have been widely used to guide the research on restorative environments: Stress Recovery Theory (SRT) [5] and Attention Restoration Theory (ART) [6]. SRT claims that restoration is derived from the release of stress and the reduction of negative moods through being exposed to restorative environments. Natural environments are more conducive to improving physical and mental health than urban environments. ART distinguishes two types of attention: directed attention and involuntary attention. When individuals were in a state of tension for a long time, directed attention would be consumed, which resulted in mental fatigue. Exposure to natural settings is believed to be a good way for directed attention restoration. ART proposed four specific components that a restorative environment should have: Being away is a feeling of being distant from everyday routine; Fascination implies a setting containing landscape elements that hold one’s attention without any effort; Coherence can take place in an environment which is well-organized; Compatibility signifies the match between the environment and what the person is expected to do in the environment. Hartig [7] designed the Perceived Restorativeness Scale (PRS) to measure the restorative potential of an environment based on the four specific components distinguished in ART. The PRS has been developed by many researchers [8,9] and widely used in several studies [10,11].

Urban natural environments take the form of urban green spaces (e.g., urban forests, parks, street trees) and blue spaces (e.g., ponds, lakes) [12]. Some studies have shown that interacting with urban natural environments can relieve the decline of physical and cognitive functioning and improve mental health and well-being of the elderly [13,14,15]. However, simply understanding the restorative values of natural environments is not enough to provide an essential guideline for landscape planning and design in practice [16]. For landscape planners and designers, it is necessary to identify the specific characteristics of landscape that are the most popular and the most mental restorative for humans, particularly the elderly [17]. To address this concern, in recent years, some studies have intended to categorize the specific characteristics of urban natural environments for human well-being and health from the point of view of the public’s experience [18,19]. The experienced qualities in urban natural environments can be subdivided into different “perceived sensory dimensions.” One such classification system has been developed over the last 40 years by researchers from the Swedish University of Agricultural Sciences and the University of Copenhagen [20]. The latest version distinguishes the following eight perceived sensory dimensions (PSDs): Serene, Nature, Rich in species, Space, Prospect, Refuge, Social and Culture (Table 1). The eight PSDs have been widely used in the studies of landscape assessment and planning, as well as environmental restoration. Grahn and Stigsdotter indicated that the environments with more qualities of the Refuge, Nature, and Rich in species dimensions, and less qualities of the Social dimension would be more restorative for stressed individuals in the Scandinavian cultural context [20]. Peschardt and Stigsdotter found that Serene and Social dimensions were closely associated with people’s perceived restorativeness in small, public urban green spaces [21]. Given the validity of the eight PSDs for guiding the design of restorative environments, the eight PSDs were thus applied as a tool in this study to examine the restorative environment for the elderly in China. Its specific objectives were to investigate:

1. Representation of the eight PSDs in different types of urban environment as perceived by the elderly.
2. Differences in preference and psychological restoration of the elderly among the different types of urban environment.
3. Relationships between the number of highly perceived PSDs, preference and psychological restoration.
4. Relationship between the eight PSDs and psychological restoration in the different types of urban environment.

## 2. Materials and Methods 

### 2.1. Study Area

**Figure 1:**
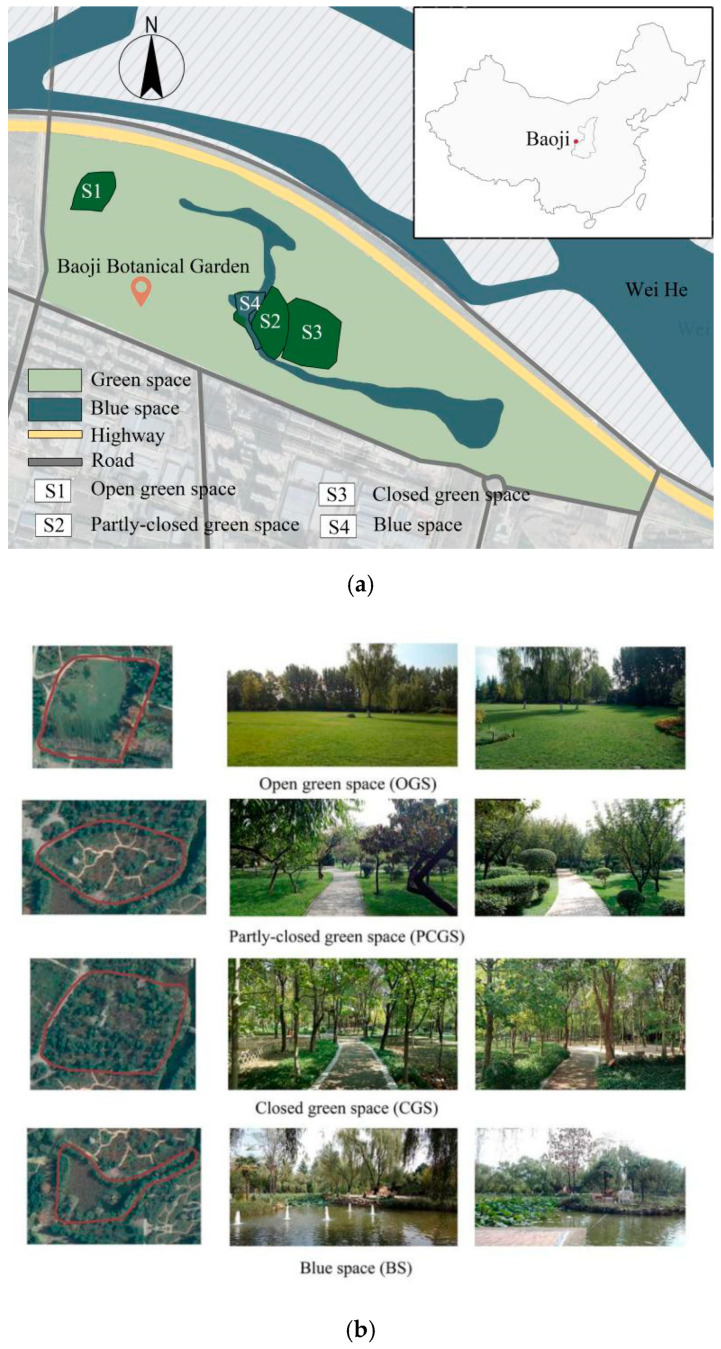
Study area and photographs of the four study sites (OGS: Open green space; PCGS: Partly-closed green space; CGS: Closed green space; BS: Blue space). (**a**) Study area; (**b**) Eight photographs of the four study sites.

Since bringing people in the field to evaluate an actual landscape is costly and time consuming [22], the on-site survey was replaced by photo elicitation utilized as visual stimuli, which is relatively more convenient and feasible [23]. Baoji Botanical Garden, located in Baoji city (China), was selected as the study area for collecting photos. It was established in 1979 and covers an area of 70.3 ha in total with approximately 40 ha for recreation, which includes 4.7 ha of water surface. Today, it has become one of the most popular recreational gardens in Baoji, in which the landscape types are diverse and representative (Figure 1a). After a visual and bio-physical field investigation in the garden by the experts of landscape architects, four types of natural environment with distinct land cover type and canopy cover ratios of trees and shrubs were determined as shooting sites, including open green space (OGS), partly-closed green space (PCGS), closed green space (CGS) and blue space (BS) (Table 2). Two photos were taken in each site from different directions at the average eye level (*h* = 1.6 m) on sunny and windless days in September, 2019. Eight photos were finally obtained and printed on A4 hard photo paper in true color to be used as visual stimuli (Figure 1b).

### 2.2. Participants

Respondents were randomly selected among adults who were using open/green spaces in their residential areas and informed about the survey’s objectives and answering procedure. Those willing to participate were then given the questionnaire and invited to fill it in during their stay in the area. The selection criteria of the participants were (1) adults 60 years old or over; (2) adults with no cognitive and communication difficulties. An initial total of 309 older people were recruited for the survey and 9 were excluded due to incomplete questionnaires. Thus, a final total of 300 participants were included based on the perceptions of the photos representing the four types of environments. All subjects gave their informed consent for inclusion before they participated in the study. The study was conducted in accordance with the Declaration of Helsinki, and the protocol was approved by Ethics Committee of College of Landscape Architecture and Arts, Northwest A&F University. 

### 2.3. Data Collection

Compared with interviews, the questionnaire survey was conducted instead in this study due to collecting large amount of information in a short period of time and in a relatively cost effective way, and being analyzed more scientifically and objectively. The questionnaire consisted of four parts. The first part contained the demographic information of the respondents including gender and age. The second part focused on the perception of the eight PSDs. The perceived level of each dimension was measured on a 5-point Likert scale ranging from 1 = “none or very weak” to 5 = “very strong.” The third part included a question regarding the preference for the selected environments and reasoning for the preference levels. Using a 5-point Likert scale to assess the preference level (ranging from 1 = “extremely dislike” to 5 = “extremely like”). The fourth and final part of the questionnaire evaluated the psychological restoration of the respondents using the Perceived Restorativeness Scale (PRS). The scale included 16 items: 2 items for Being away, 5 items for Fascination, 4 items for Coherence and 5 items for Compatibility. Each item was accurately translated in Chinese and assessed by a 5-point Likert scale ranging from 1 = “not agree at all” to 5 = “agree very much.” Each participant was asked to perceive only one type of the selected environments, which resulted in two photos being evaluated by each participant. The average time of the visual stimuli and questionnaire was approximately eight minutes in total for each participant.

### 2.4. Data Analysis

All statistical analyses were carried out using the SPSS 20 software (IBM, Armonk, NY, USA). Preceding the analysis, the reliability of perceived restorativeness scores was calculated. Cronbach’s alpha was 0.958, indicating a good internal consistency and reliability. To identify which PSDs were frequently perceived and which were seldom perceived by the elderly in each type of urban environment, an analysis of arithmetic means and ANOVA with post hoc tests were used. According to Tosun [24], the mean value of the 5-point Likert scale of the eight PSDs can be divided into three levels to indicate the level of perception: low (1–2.5), medium (2.5–3.5) and high (>3.5). One-way ANOVA with post hoc tests was conducted to analyze the differences in landscape preference and psychological restoration among the four types of urban environment. The mean values of the items of each restorative component were used to identify the restorative effect in this component, and the mean values of all the 16 items were used to identify the overall psychological restoration of each participant. The correlation analyses between landscape preference, psychological restoration and the number of highly perceived PSDs were conducted. To explore which PSDs were associated with preference and psychological restoration of the elderly within different types of urban environment, a stepwise multiple liner regression analysis was conducted, using the four restorative components, overall restoration and preference scores as the dependent variable respectively, and the eight PSDs as the independent variables.

## 3. Results

### 3.1. Demographic Characteristics of the Respondents

**Figure 2:**
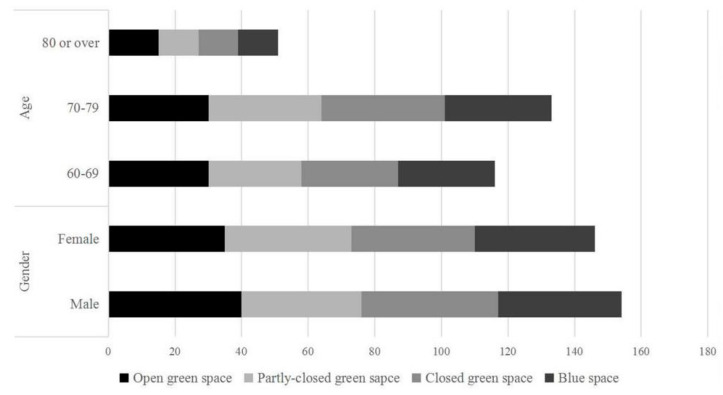
Demographic characteristics of the respondents.

A total of 300 respondents were included in the study. The results showed that the number of male and female participants was relatively equal (51% were male, 49% were female), and most of the participants were aged around 60–79 (83%). The participants were evenly distributed in the four types of the selected environments (Figure 2).

### 3.2. Representation of the Eight PSDs in the Selected Urban Environments

The respondents were generally able to understand the eight PSDs well. The results showed that older people most frequently perceived the Serene dimension, followed by Refuge, Prospect, Rich in species, Culture and Nature. Space, followed by Social, were the least perceived dimensions. According to the ANOVA results, respondents had significant differences in perception of the eight PSDs in the four types of urban natural environments. Prospect was the only sensory dimension that was highly perceived in OGS. Serene and Refuge were the sensory dimensions that were highly perceived in PCGS. Nature, Serene, Refuge and Culture were the most strongly experienced in CGS, and Serene, Rich in species, Culture, Nature, Prospect and Refuge were highly perceived in BS (Table 3).

### 3.3. Differences in Preference and Psychological Restoration among Different Types of Urban Environment

#### 3.3.1. Differences in Landscape Preference among Different Urban Environments

**Figure 3:**
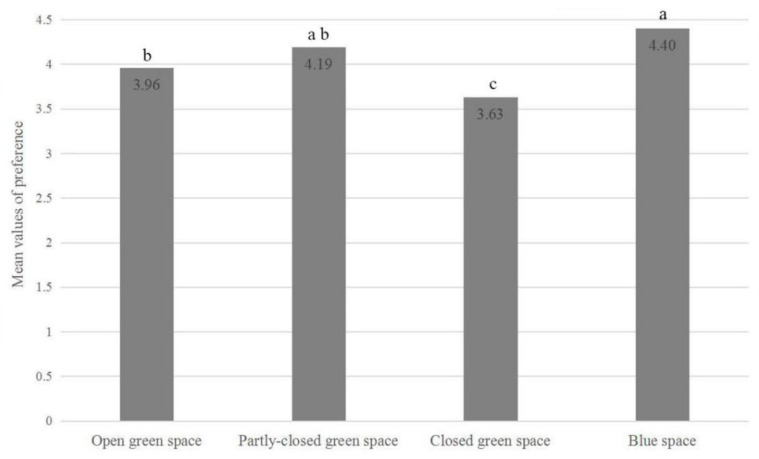
ANOVA with a post hoc test on differences in preference among the four study sites (significant difference at the 0.05 level is shown by different letters a, b and c).

The results of the one-way ANOVA indicated that the participants had significant differences in preference for different types of urban natural environments (F = 19.64, *p* < 0.01). BS had the highest preference score (M = 4.40, SD = 0.66), followed by PCGS (M = 4.19, SD = 0.57) and OGS (M = 3.96, SD = 0.67). CGS was least preferred by the respondents (M = 3.63, SD = 0.69) (Figure 3).

#### 3.3.2. Differences in Perceived Restorativeness among Different Urban Environments

**Figure 4:**
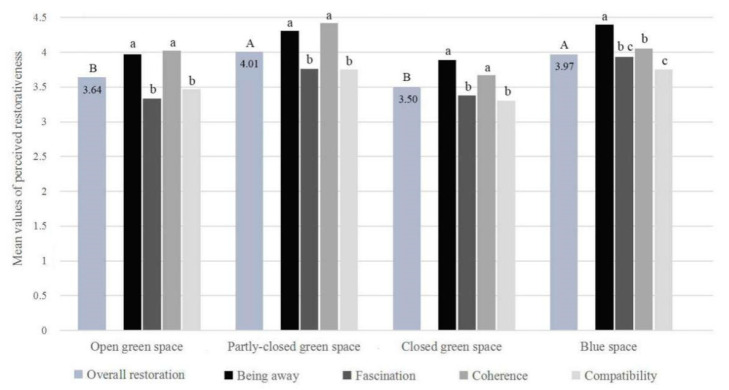
ANOVA with post hoc tests on differences in perceived restorativeness among the four study sites (Significant differences in overall restoration at the 0.05 level is shown by different letters A and B; significant differences in the four restorative components at the 0.05 level is shown by different letters a, b and c).

A one-way ANOVA showed that the participants significantly differed in the overall restoration experienced among the four urban natural environments (*F* = 20.95, *p* < 0.01). PCGS and BS were perceived as the most restorative environments, while OGS and CGS were less perceived (Figure 4).

The results also indicated that in OGS, PCGS and CGS, Being away and Coherence were more perceived, while Fascination and Compatibility were less perceived. In BS, Being away was most perceived, followed by Coherence and Fascination, while Compatibility was the least perceived. Overall, in the four study sites, Being away and Coherence were more perceived, while Fascination and Compatibility were less perceived (Figure 4).

### 3.4. Relationship between the Number of Highly Perceived PSDs, Preference and Psychological Restoration

**Figure 5:**
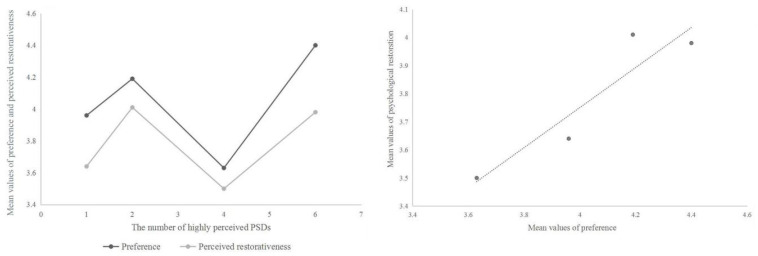
The relationship between the number of highly perceived PSDs, preference and psychological restoration.

BS had the most number of sensory dimensions that were highly perceived (*n* = 6), followed by CGS (*n* = 4). There were 2 sensory dimensions that were highly perceived in PCGS, and only 1 sensory dimension was highly perceived in OGS (Table 4). The correlation analysis showed that there was no significant correlation between preference and the number of highly perceived PSDs (*R* = 0.308, *p* = 0.692), as well as between psychological restoration and the number of highly perceived PSDs (*R* = 0.219, *p* = 0.781). However, a significant positive correlation was shown between landscape preference and psychological restoration (*R* = 0.785, *p* < 0.01). This indicated that the more a person prefers a landscape, the more psychological restoration he or she will perceive in this landscape and vice versa (Figure 5).

### 3.5. Relationship between the PSDs and Psychological Restoration in the Different Types of Urban Environments

The associations between the PSDs and psychological restoration in the different landscape types showed that Serene, Prospect and Social were the three dominating PSDs in OGS that had a significant effect on overall restoration. The Culture dimension had a significant predictive effect on Compatibility. In PCGS, the Serene and Refuge dimensions were two significant predictors for psychological restoration. Nature, however, had a significant negative effect on Coherence. As for CGS, the Space and Refuge dimensions had significant positive effects on psychological restoration, while the Nature dimension had a negative effect. In terms of BS, Serene, Prospect and Refuge dimensions had significant predictive effects on psychological restoration. Nature had a significant positive effect on Fascination, and Social had a positive effect on Compatibility (Table 5).

## 4. Discussion

### 4.1. Urban Natural Environments that Were More Preferred and More Restorative for the Elderly

In the present study, the results indicate that BS was most preferred by the elderly, and also had a higher restorative potential. These findings are consistent with previous studies which claimed that water is an important element for increasing the attractiveness and restorative potential of an environment [25,26,27,28]. This could be explained by the fact that according to an evolutionary perspective, human beings naturally have a positive response to aquatic elements for seeking tranquility and healing [29]. For the elderly, broad water surfaces or beautiful water features make them feel more alive and satisfy their desire for nature [15], as some respondents commented that “Water makes me feel quiet and calm,” and “Blue space is a good place to relax.” 

As for the urban green spaces, PCGS was more preferred and more restorative for the elderly than OGS and CGS. Many studies have shown that plants are an important element in improving people’s landscape preferences and restoration [30,31,32]. According to SRT and ART, restorative environments should have certain structures and natural elements, which could satisfy the four restorative components [5,6]. Plants can enable a setting configuration to be more complex and diverse, which enhances the scenic beauty and attraction of the environment, and also creates a calm and private place for the elderly to take a break and enjoy themselves [33,34]. Many respondents’ comments concerning OGS were “There are few plants in it,” “Trees are not enough for me,” “This environment is monotonous and not attractive,” while PCGS was more preferred by the elderly because “The environment has many plants,” “The vegetation is rich,” and “The plants are attractive.” However, in this study, CGS containing a large amount of trees was least preferred and restorative, even less so than OGS. This result was inconsistent with some studies indicating that green spaces with more trees or higher vegetation density were more preferred or more restorative [34,35,36]. This may be because for the elderly, environmental safety is a crucial factor when experiencing green spaces [37,38]. When vegetation density is too high, it could give older people a sense of insecurity, which might affect the psychological restoration of the elderly. Moreover, too many trees and shrubs would also make the environment untidy, thus reducing the aesthetics of the environment [39]. For example, many respondents considered CGS as “depressing,” “too many trees,” and “messy.”

### 4.2. Relationship between the Number of Highly Perceived PSDs, Preference and Psychological Restoration

In this study, the results indicated that the number of highly perceived PSDs did not significantly correlate with preference and psychological restoration of the elderly, which suggests that an environment with greater numbers of highly perceived PSDs may be neither preferred by, nor more restorative for the elderly. This finding could be explained by the fact that not all of the PSDs are strongly associated with public preference and restoration [40]. If PSDs that were highly rated in an environment did not have significant positive effects on preference and restoration, or even had negative effects, preference and restorative potential of the environment would not increase. Therefore, for landscape design, the specific PSDs which positively affect preference and restoration should be focused on rather than more PSDs be created in a certain environmental setting.

The results also suggested that older adults’ preference for green space had a significant positive correlation with their psychological restoration, which is consistent with previous findings [34,41,42]. Several studies have assumed that preference is reflective of people’s perception and assessment of environments [43]. People are more likely to prefer an environment wherein they can feel comfortable and relaxed, and this kind of environment is also conducive to psychological restoration [44]. Therefore, landscape preference can be an important reference factor in the construction of restorative environments.

### 4.3. The PSDs That Were Associated with Psychological Restoration of the Elderly in Different Environments

The results indicated that there was a significant relationship between certain PSDs and psychological restoration, and the PSDs that had significant predictive effects on psychological restoration varied across different types of environments. These findings support a previous study which found that the effects of the same PSDs varied with different environments due to different landscape characteristics and configurations [45].

PCGS was the most restorative green space for the elderly, with Refuge and Serene dimensions being two significant predictors for restoration. This result was in line with the previous finding that high-stressed people preferred more *Serene* [46]. Jensen [47] showed that quietness is one of the main reasons why people like natural environments. Noise in urban environments has a great impact on the physical and mental health of the elderly [48], while a silent and calm environment can help the elderly reduce stress and mental fatigue. Moreover, a secluded and enclosed place where people can do what they like without being disturbed is also an important requirement [49]. Serene and Refuge are two important dimensions that contain characteristics that can meet these needs well. It could also be found from the results that Serene and Refuge were the two sensory dimensions most strongly perceived by the elderly in PCGS, which indicated that the partly-closed vegetation structure could create a quiet, private and safe environment to satisfy the needs of the elderly. This might be the reason for the highest restorative effect of PCGS. Therefore, with regard to the construction of PCGS, the characteristics of the Serene and Refuge dimensions should be given priority when considering the design of the environment. In addition, the dimension of Nature should not be created more due to its negative influence on Coherence in the partly-closed green space.

BS also had a stronger restorative effect, with Serene, Prospect and Refuge dimensions positively affecting restoration. Compared with the PCGS, the Prospect dimension was also a factor affecting restoration in BS. This result supported the previous study that an environment with diverse vegetation as well as more open view is regarded as being optimal for restoration [40]. According to the prospect-refuge theory, people need a safe environment providing shelter to hide themselves as well as a clear field of vision which allows people to detect danger [49]. Therefore, a balanced level of Prospect and Refuge dimensions is closely linked to mental restoration. Older people could easily perceive Serene, Prospect and Refuge dimensions in blue space because of the open water feature and surrounding plants, which might be the reason why blue space had more restorative potential. Therefore, with regard to blue space design, Serene, Prospect and Refuge dimensions are of the most importance in designing a positive environment. In addition, Nature and Social dimensions could also be created to increase Fascination and Compatibility. 

The restorative potential of OGS was relatively lower across the four types of urban landscape due to less perceived sensory dimensions of Serene and Social, while Prospect, Serene and Social were potential predictors for mental restoration. This result could support the previous study indicating that short-cut lawns had a stronger restorative effect because it provides a variety of activities to people [50]. Social isolation has become a challenge among older people living in cities [51]. Urban green spaces provide open places for recreational, physical and social activities, which are beneficial to the mental health of the elderly [52,53]. This finding is consistent with the findings of Peschardt and Stigsdotter [21], which showed that Social and Serene dimensions were significantly associated with perceived restorativeness. Interestingly, Social seems to contradict Serene, but the Serene dimension does not equate to the absence of sound in the environment. Proper sounds of activity in the environment are often conducive to psychological restoration of people living in urban areas [21]. In the future, a balance between the Social and Serene dimensions is important in constructing OGS for the elderly. In addition, since the Culture dimension was important for Compatibility, some cultural statues or ornamental flower beds could be added in open green spaces for increasing attractiveness.

CGS was the least restorative natural environment for the elderly in our study, while Refuge and Space dimensions had positive predictive effects on restoration and the dimension of Nature had a negative effect. Space is positively associated with psychological restoration due to the provision of spacious and a sense of being free, and it is similar to Coherence, which is one of the four components that a restorative environment should have [20,21]. Interestingly, the respondents were not willing to perceive a greater *Nature* dimension in CGS, which is not in line with previous studies. An explanation for this could be that being exposed to the green spaces with a high-density vegetation cover may increase stress and mental fatigue due to feeling insecure [54,55]. Also, legibility and maintenance of green spaces are crucial factors for older adults [56,57], while environments that are too natural or wild might, to some extent, reduce the legibility, and would make the environment untidy and messy, thus reducing restorative values. The dimension of Nature was strongly perceived in the selected CGS, while Space was not highly perceived, which might affect the restorative potential of the closed green space. In the future, moderate landscape management should be implemented to form an orderly layout combining rarefaction and density of vegetation cover in the closed green space.

### 4.4. Limitations and Future Research

There are still some limitations in the study. Firstly, some research claimed that factors such as socio-demographic variables, health condition and the use of natural environment (e.g., type of activity) may influence people’s preference or restoration [58,59,60]. Therefore, older adults with different backgrounds should be considered in the future in order to get more extensive conclusions. Second, the present study only selected four types of urban natural environments. A greater diversity of urban environments should be examined. Moreover, the physiological restoration of the elderly in the different types of urban environments needs to be explored in the future.

## 5. Conclusions

This study applied the eight PSDs as a tool to explore the specific characteristics of urban natural environments that were the most liked and the most mentally restorative for the elderly in China. The results indicated that older people had significant differences in perception of the eight PSDs in different urban natural environments. Blue space and partly-closed green space were more preferred by the elderly, and also had more psychological restorative effects on the elderly. The findings of the study can be used to produce a construction proposal of urban restorative environments for the elderly in the future: partly-closed green space with more characteristics of the Serene and Refuge dimensions, and blue space with more Serene, Refuge and Prospect characteristics were optimal landscape qualities for psychological restoration of the elderly. In addition, open green space with more Prospect, Serene and Social properties, and closed green space with more Space, Refuge and less Nature properties could also increase the psychological restoration of older adults. These results can be used for the elderly-oriented restorative environment design in practice.

## Figures and Tables

**Table 1:** Brief descriptions of the eight PSDs (perceived sensory dimensions).

Number	Perceived Sensory Dimensions	Key Qualities and Features
1	Serene	The environment is silent and calm; No contact with many people; Not disturbed by traffic noise.
2	Nature	An environment with natural qualities; Wild and untouched; Free growing.
3	Rich in species	Various species of plants; Various species of animals, such as birds, insects, etc.
4	Space	A spacious and free environment; A lot of trees; Not crossed by too many roads and paths.
5	Prospect	An environment with open views; Vistas over the surroundings; Plane and well-cut grassy surfaces.
6	Refuge	An environment with many bushes; One can sit and watch other people being active.
7	Social	An abundance of people and movements in the environment; Possible to watch entertainment or exhibitions.
8	Culture	Many cultural features, such as fountains, statues, etc; Decorated with ornamental plants or flowers.

**Table 2:** Classification of urban natural environments.

Level 1	Level 2	Characteristics of Each Environment
Green space (GS)	Open green space (OGS)	<30% canopy cover of trees/shrubs
	Partly-closed green space (PCGS)	30%–70% canopy cover of trees/shrubs
	Closed green space (CGS)	>70% canopy cover of trees/shrubs
Blue space (BS)	-	Open lake (dominated by water with certain greenery)

**Table 3:** Arithmetic means of perception of the eight PSDs in different landscape types.

PSDs	OGS		PCGS		CGS		BS		Total of Mean	Total of Rank
	Mean	MD	Mean	MD	Mean	MD	Mean	MD		
Serene	3.13	1.17 **	**3.82**	Reference	**3.82**	0.21	**3.78**	Reference	3.64	1
Refuge	1.75	2.57 **	**3.54**	0.28	**3.71**	0.32 *	**3.53**	0.25	3.13	2
Prospect	**4.31**	Reference	2.73	1.09 **	1.74	2.28 **	**3.52**	0.26	3.06	3
Rich in species	2.04	2.27 **	2.85	0.97 **	3.37	0.65 **	**3.77**	0.01	3.01	4
Culture	2.15	2.16 **	2.55	1.27 **	**3.53**	0.50 **	**3.75**	0.03	2.99	5
Nature	1.64	2.76 **	2.55	1.57 **	**4.03**	Reference	**3.56**	0.22	2.88	6
Space	2.85	1.45 **	2.43	1.39 **	3.33	0.69 **	2.51	1.27 **	2.79	7
Social	3.09	1.21 **	2.19	1.64 **	2.23	1.79 **	2.96	0.82 **	2.61	8

The bold numbers indicate a high degree of respondents’ perception. * The significance of difference is at the 0.05 level. ** The significance of difference is at the 0.01 level.

**Table 4:** The three-scale levels of perception of the eight PSDs.

Landscape Type	Level of Perception of the Eight PSDs in the Four Urban Natural Environments		
	High	Medium	Low
OGS	Prospect	Serene/Space/Social	Refuge/Rich in species/Culture/Nature
PCGS	Serene/Refuge	Prospect/Rich in species/Culture/Nature	Space/Social
CGS	Serene/Refuge/Culture/Nature	Rich in species/Space	Prospect/Social
BS	Serene/Refuge/Prospect/ Rich in species/Culture/Nature	Space/Social	None

**Table 5:** The PSDs that had significant effects on psychological restoration.

Perceived Restorativeness	OGS	PCGS	CGS	BS
Being away	Serene/Prospect/Social	Serene	Space/Nature */Refuge	Serene/Refuge
	(Adjusted R^2^ = 0.374)	(Adjusted R^2^ = 0.235)	(Adjusted R^2^ = 0.575)	(Adjusted R^2^ = 0.331)
Fascination	Serene/Prospect	Serene/Refuge	Space/Nature */Refuge/Social	Serene/Prospect/Nature
	(Adjusted R^2^ = 0.248)	(Adjusted R^2^ = 0.580)	(Adjusted R^2^ = 0.463)	(Adjusted R^2^ = 0.314)
Coherence	Serene/Prospect/Social	Refuge/Nature *	Space/Nature *	Serene/Prospect
	(Adjusted R^2^ = 0.323)	(Adjusted R^2^ = 0.403)	(Adjusted R^2^ = 0.331)	(Adjusted R^2^ = 0.390)
Compatibility	Serene/Prospect/Culture	Serene/Refuge	Space/Nature */Refuge	Serene/Refuge/Prospect/Social
	(Adjusted R^2^ = 0.419)	(Adjusted R^2^ = 0.634)	(Adjusted R^2^ = 0.596)	(Adjusted R^2^ = 0.534)
Overall restoration	Serene/Prospect/Social	Serene/Refuge	Space/Nature */Refuge	Serene/Refuge/Prospect
	(Adjusted R^2^ = 0.433)	(Adjusted R^2^ = 0.687)	(Adjusted R^2^ = 0.705)	(Adjusted R^2^ = 0.540)

* The predictive effect is negative.

## Data Availability

The data presented in this study are available on request from the corresponding author. The data are not publicly available due to policy of the institute.
